# Bread

**Published:** 1878-11

**Authors:** 


### BREAD.

It is said that one of the most wholesome kinds of bread that can be used is made thus, without salt, saleratus, yeast, or rising of any sort.

Take bolted or unbolted flour or meal, thoroughly moisten the whole with pure soft water, scalding hot, that is, about one hundred and sixty degrees Fahrenheit, make it up firm, not sticky, then roll and cut into strips, or any other form, not over a quarter of an inch thick, and half an inch broad. Bake quickly in a hot oven until the dough has acquired a soft, fine, brown color, or until the water has nearly all evaporated.

Hydropathists sav that a sweeter bread than this was never tasted

